# Low grade squamous intra-epithelial lesions and human papillomavirus infection in Colombian women

**DOI:** 10.1038/sj.bjc.6600650

**Published:** 2002-12-02

**Authors:** M Molano, A J C van den Brule, H Posso, E Weiderpass, M Ronderos, S Franceschi, C J L M Meijer, A Arslan, N Munoz, and HPV Study Group Group

**Affiliations:** Department of Pathology, Vrije Universiteit Medical Centre, De Boelelaan 1117, 1081 HV, Amsterdam, The Netherlands; Division de Investigacion, Instituto Nacional de Cancerologia, Bogota, Colombia; Unit of Field and Intervention Studies, International Agency for Research on Cancer, Lyon, France

**Keywords:** LSIL, HPV, *C. trachomatis*, Colombia, risk factors

## Abstract

Low grade squamous intra-epithelial lesions could be considered as a manifestation of human papillomavirus exposition, however the discrepancy between rates of infection with human papillomavirus and development of low grade squamous intra-epithelial lesions is notable. Here we report a cross-sectional three-armed case–control study in the Colombian population, to compare the risk factors of women with low grade squamous intra-epithelial lesions with that of human papillomavirus DNA-negative and positive women with normal cytology.

*British Journal of Cancer* (2002) **87**, 1417–1421. doi:10.1038/sj.bjc.6600650 www.bjcancer.com

© 2002 Cancer Research UK

## 

Cervical cancer, the main cause of death from cancer among Colombian women, is known to be preceded by a series of squamous intra-epithelial lesions (SIL). Recently it has been established that human papillomavirus (HPV) is not only the central cause of cervical cancer, but also a necessary cause (Walboomers *et al*, 1999).

Natural history studies indicate that the chain of events leading to cervical cancer starts with HPV infection, followed by high-grade squamous intra-epithelial lesions (HSIL) and cancer. The category HPV infection could include a range of diagnoses from HPV DNA detectable only by PCR without cytological abnormalities (latent HPV infection) to CIN-1 lesions indistinguishable from koilocytic atypia, called low-grade squamous intra-epithelial lesions (LSIL) or productive HPV infection. LSIL should then be considered as a sign of exposure to HPV.

It is currently unclear if the determinants of HPV DNA detection in women with normal cytology are the same or similar to those of LSIL. A recent follow-up study in young women suggests that risk factors for HPV infection are different from those of LSIL (Moscicki *et al*, 2001).

In a previous study the risk factors for HPV DNA detection among Colombian women with normal cytology were identified (Molano *et al*, 2002). We report here a cross-sectional three-armed case-control study in the same population, to compare the risk profile of women with LSIL lesions with that of HPV DNA-negative and positive women with normal cytology.

## MATERIALS AND METHODS

### Study population

This study was conducted in Bogota, Colombia. Briefly, between 1993 and 1995 the Colombian Cancer Institute conducted a census in four health districts in Bogota. The first 2000 women aged 18–85 years identified in the census were invited to participate. In addition, 200 adolescents aged 13–17 years consulting an adolescent clinic giving contraceptive counselling were also invited to participate. At recruitment, all women were interviewed by specially trained interviewers using a structured questionnaire on socio-demographic characteristics, lifelong sexual behaviour, reproductive history, smoking and dietary habits. After interview all women underwent a pelvic examination during which cervical scrapes were collected for routine Pap smear and HPV DNA and *Chlamydia trachomatis* detection.

Out of the 2200 invited women, 53 refused to participate, eight were considered ineligible (history of hysterectomy or cervical cancer, mental illness), 14 did not fill in the epidemiological questionnaire, 29 did not provide cervical scrapes and 1010 had inadequate cervical specimens for amplification, as measured by failure to amplify a human β-globin fragment. Thus 1995 (90.7%) women with a cytological diagnosis and with adequate cervical samples for HPV DNA and *C. trachomatis* PCR testing constitute the sampling frame for the study. Informed consent was obtained from all women and the ethical committees of the National Cancer Institute of Colombia and of the IARC cleared the study protocol.

### Case-control selection

The smears taken at recruitment were read by two expert cytopathologists as follows: normal cytology in 1845 women (92.5%), LSIL in 70 (3.5%) women, atypical squamous cells of undetermined significance (ASCUS) in 30 (1.5%), atypical glandular cells of undetermined significance (AGUS) in 14 (0.7%), HSIL in 8 (0.4%), invasive cervical cancer in 2 (0.1%) and 26 (1.3%) had inadequate Pap smears.

Cases for the case–control analysis were those with a diagnosis of LSIL (two cases diagnosed HSIL at cytology, but classified LSIL at histology are included. On the other hand, two cases first classified as LSIL at cytology were diagnosed HSIL and cancer *in situ* at histology and were excluded). Controls were selected from the pool of 1845 women with normal cytology. Eight controls per case with LSIL diagnosis were selected, matched for age (±2 years) and date of recruitment (±6 months), giving a total of 557 control women. Two subgroups of control women were compared to the cases: one of 459 HPV DNA-negative and another of 98 HPV DNA-positive women.

### HPV DNA detection and genotyping

HPV DNA detection in the samples was performed previously by a standard GP5+/GP6+ PCR EIA-based assay (de Roda Husman *et al*, 1995). Briefly, HPV-positive samples were subjected to EIA HPV group-specific analysis using cocktail probes for high- and low-risk HPVs (Jacobs *et al*, 1997). The high-risk HPV cocktail probe consisted of oligoprobes for HPV 16, 18, 31, 33, 35, 39, 45, 51, 52, 56, 58, 59, 66 and 68 and the low-risk HPV consisted of oligoprobes for HPV 6, 11, 40, 42, 43, 44, HPV 82 (MM4), (Iso 39) HPV 83 (MM7), HPV 84 (MM8), HPV 71 (CP8061), HPV 81 (CP8304), HPV 26, 34, 53, 54, 55, 57, 61, 70, 72 and 73. Additionally, HPV positivity was assessed by Southern blot hybridisation of GP5+/GP6+ PCR products with the general probe of specific [α-^32^P]dCTP-labelled fragments from cloned DNA of HPV 6, 11, 16, 18, 31, and 33 (van den Brule *et al*, 1990; de Roda Husman, 1995). Samples which were positive by Southern blot analyses and negative by high-risk/low-risk EIA were considered as HPV X or undetermined type.

### *Chlamydia trachomatis* detection by PCR

The detection of *C. trachomatis* was performed as described previously (Morré *et al*, 1998). Plasmid endogenous-specific biotinylated primers were used for PCR amplification. The biotinylated PL6.1/PL6.2 PCR products were detected using an enzyme immunoassay as described previously (Jacobs *et al*, 1997; Munk *et al*, 1999). As a positive control, a 10-fold dilution series of *C. trachomatis* L2 DNA was used as previously described (Morré *et al*, 1996), resulting in a detection sensitivity corresponding to 0.01–0.1 inclusion forming units (IFU).

### Statistical analysis

Odds ratios (ORs) and 95% confidence intervals (CIs) were estimated using unconditional logistic regression. Two separate analyses were performed. The first, analysing risk factors for the development of LSIL, compared LSIL (all) to a matched sample of cytology controls. The second compared LSIL-positive for HPV DNA with two subgroups of normal cytology controls, one including only HPV DNA-negative controls, the other including only HPV DNA-positive controls.

The ORs estimates were adjusted for age (grouped as <20, 20–24, 25–29, 30–34, 35–39, 40–44, 45–54, ⩾55).

## RESULTS

The estimated overall HPV DNA prevalence in the total study population from which we selected our three study groups was 16.5%. Table 1

**Table 1:**
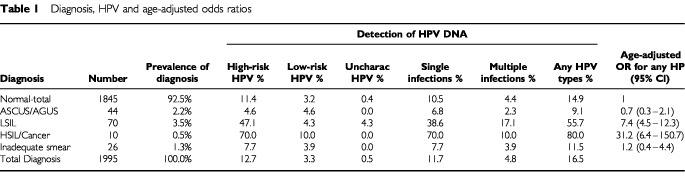
Diagnosis, HPV and age-adjusted odds ratios

presents the distribution of study subjects, the prevalence of HPV DNA by diagnostic category and the age-adjusted ORs for any type of HPV. More severe lesions were associated with higher prevalence of HPV DNA, higher prevalence of high-risk HPV types and higher ORs. No association with HPV was observed for the group diagnosed as ASCUS/AGUS.

Table 2

**Table 2:**
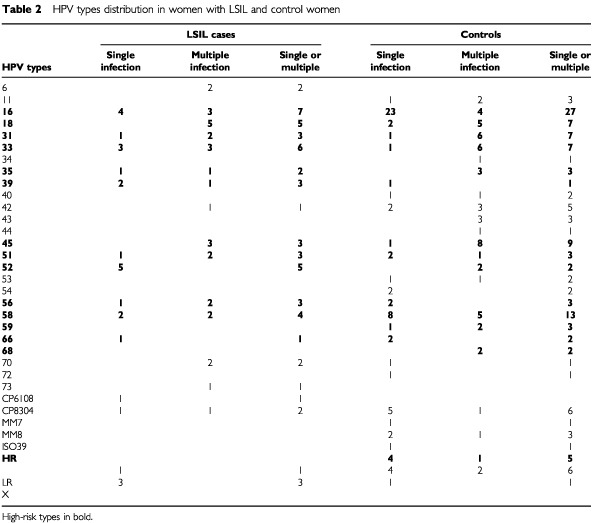
HPV types distribution in women with LSIL and control women

shows the distribution of the HPV types detected in 70 women diagnosed with LSIL and in 557 control women. Eighteen different types were detected in LSIL and 30 among controls. HPV 6, 73 and CP6108 were exclusively found in LSIL and HPV types 11, 34, 40, 43, 44, 53, 54, 59, 68, 72, MM&, ISO39 and MM8 were exclusively detected among controls. The most common type detected in both cases and controls was HPV 16. All women with multiple infections (except one) had at least one high-risk type and the prevalence of these multiple infections among HPV-positive women was similar in LSIL (30.8%) and controls (27.6%).

The age-adjusted OR for LSIL associated with single HPV infections was 6.1 (95% CI=3.4–10.9) while that for multiple infections was 7.6 (95% CI=3.4–16.9). The median age of women with LSIL was 30.8. The prevalence of this diagnosis was higher in women younger than 25 years where it was 4.4% and then decreased to 1.7% in women over 55–64 years old and 0.0 in women over 65. The prevalence of *C. trachomatis* was 7.1% in the overall LSIL group and 5.2% in the control group (Table 3

**Table 3:**
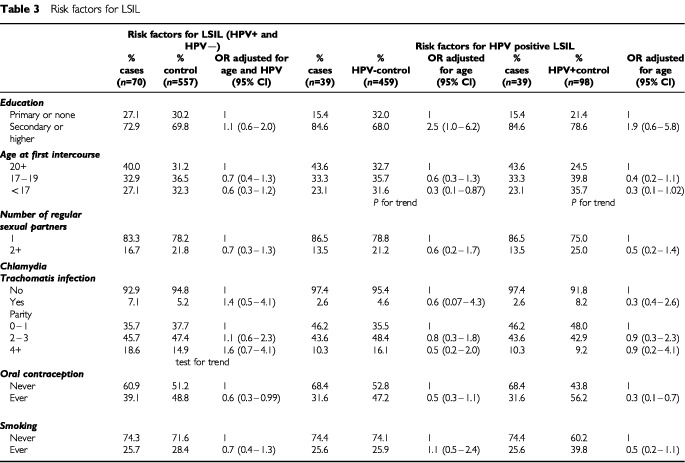
Risk factors for LSIL

).

When all LSIL cases (HPV-positive and HPV-negative) were compared with all controls (HPV-positive and negative) and ORs adjusted for age and HPV were estimated, a significantly reduced risk appeared with ever use of OCs. Non-significant increased risk associated with the detection of *C. trachomatis* and non-significant decreased risks with number of partners, age at first sexual intercourse and smoking were observed (Table 3). When women with HPV DNA-positive LSIL were compared with HPV DNA-negative controls (Table 3), a significant increased risk for women attaining higher educational level was observed, and the reduced risk with number of partners, age at first sexual intercourse and with ever use of OCs persisted, however non-significantly. When HPV DNA-positive LSIL was compared with HPV DNA-positive controls, the reduced risk associated with the use of OCs was significant (OR=0.3; 95% CI=0.1-0.7), while the association with higher education lost statistical significance, and non-significant decreased risks with age at first sexual intercourse, number of partners, current infection with *C. trachomatis* and smoking were observed.

## DISCUSSION

We report here the prevalence of cervical neoplasia and of HPV types in a Colombian high-risk population.

The estimated overall prevalence of HPV was 16.5%. Among women with normal cytology it was 14.9%, while in LSIL it was 55.7%. Under the premise that HPV is a necessary cause of cervical cancer, we should expect that all true pre-neoplastic lesions contain HPV DNA. Thus, the 44.3% of HPV-negative LSIL could represent errors of diagnosis (false LSIL diagnosis), low sensitivity of the HPV assay or both. Considering that the PCR-based assays used in this study have shown excellent performance in previous investigations, the possibility of a misdiagnosis of LSIL is more likely. In line with this possibility is the fact that the prevalence of LSIL in this Colombian population (3.5%) was higher than the one reported in another high-risk population in Costa Rica (2.2%) using more strict diagnostic criteria (Herrero *et al*, 2000). The HPV DNA prevalence among women with normal cytology (14.9%) is similar to that reported from other high-risk populations in Mexico (14.5%) (Lazcano *et al*, 2001) and in previous studies carried out in Cali, Colombia (13%) (Muñoz *et al*, 1996a).

To assess the determinants of LSIL we carried out a three-armed case–control analysis in which all LSIL cases (HPV-positive or HPV-negative) were compared with a control group of normal cytology, independently of HPV status. Then LSIL HPV-positive cases were compared with two control groups: one composed by HPV-negative women with normal cytology and a second one of HPV-positive women with normal cytology.

Surprisingly, no association with the number of sexual partners was observed when LSIL cases were compared with HPV negative controls and a reduced risk of LSIL was associated with oral contraceptive (OC) use. These findings are in contrast to the risk factors for HPV DNA detection identified in women with normal cytology from the same study population. Molano *et al* (2002) have previously reported that both number of sexual partners and OC use were associated with an increased risk of HPV DNA detection in Colombian women with normal cytology. Our inconsistent results concerning number of sexual partners might be explained by the importance and the difficulty in assessing the role of male sexual behaviour in determining the risk of cervical neoplasia, as evidenced in previous studies in Colombia (Muñoz *et al*, 1996b).

In relation to OC use, our results are in line with those reported by Schiffman *et al* (1993) in a US population, that found a non-significantly reduced risk of CIN lesions among HPV-positive women associated with OC use, but not with those of a recent report by Moscicki *et al* (2001). They found that OC use had a protective effect for incident HPV infection, but it was not a risk factor for incident LSIL.

In contrast to our results, tobacco smoking has been identified as a risk factor for LSIL in other populations. For example, Krüger-Kjær *et al* (1998) found a positive association with smoking for both LSIL and HSIL among HPV-positive women in Denmark. Moscicki *et al* (2001) also identified smoking as a risk factor for LSIL in a cohort study of young US women.

Although our study was limited, for the small number of cases, it indicates that with the possible exception of OC, we were unable to identify any other important co-factor for the development of LSIL. An additional major limitation of our study, as well as of the vast majority of the studies of LSIL is the inaccuracy of the cytological diagnosis of LSIL. We have attempted to circumvent this limitation by restricting the analysis to HPV-positive-LSIL, but this considerably limited the power of our study.

On the other hand, studies aimed to assess cofactors for HSIL and cervical cancer have identified parity, smoking and long-term OC use as important cofactors that modulate the risk of progression from HPV infection or LSIL to HSIL and cervical cancer (Deacon *et al*, 2000; Hildesheim *et al*, 2001; Lacey *et al*, 2001; Moreno *et al*, 2002; Muñoz *et al*, 2002).
